# Performance of dose calculation algorithms from three generations in lung SBRT: comparison with full Monte Carlo‐based dose distributions

**DOI:** 10.1120/jacmp.v15i2.4662

**Published:** 2014-03-06

**Authors:** Jarkko J. Ojala, Mika K. Kapanen, Simo J. Hyödynmaa, Tuija K. Wigren, Maunu A. Pitkänen

**Affiliations:** ^1^ Department of Oncology Unit of Radiotherapy, Tampere University Hospital Tampere Finland; ^2^ Department of Medical Physics Medical Imaging Center, Tampere University Hospital Tampere Finland

**Keywords:** Monte Carlo dose calculation, Acuros, AXB, SBRT, photon beam radiotherapy

## Abstract

The accuracy of dose calculation is a key challenge in stereotactic body radiotherapy (SBRT) of the lung. We have benchmarked three photon beam dose calculation algorithms — pencil beam convolution (PBC), anisotropic analytical algorithm (AAA), and Acuros XB (AXB) — implemented in a commercial treatment planning system (TPS), Varian Eclipse. Dose distributions from full Monte Carlo (MC) simulations were regarded as a reference. In the first stage, for four patients with central lung tumors, treatment plans using 3D conformal radiotherapy (CRT) technique applying 6 MV photon beams were made using the AXB algorithm, with planning criteria according to the Nordic SBRT study group. The plans were recalculated (with same number of monitor units (MUs) and identical field settings) using BEAMnrc and DOSXYZnrc MC codes. The MC‐calculated dose distributions were compared to corresponding AXB‐calculated dose distributions to assess the accuracy of the AXB algorithm, to which then other TPS algorithms were compared. In the second stage, treatment plans were made for ten patients with 3D CRT technique using both the PBC algorithm and the AAA. The plans were recalculated (with same number of MUs and identical field settings) with the AXB algorithm, then compared to original plans. Throughout the study, the comparisons were made as a function of the size of the planning target volume (PTV), using various dose‐volume histogram (DVH) and other parameters to quantitatively assess the plan quality. In the first stage also, 3D gamma analyses with threshold criteria 3%/3 mm and 2%/2 mm were applied. The AXB‐calculated dose distributions showed relatively high level of agreement in the light of 3D gamma analysis and DVH comparison against the full MC simulation, especially with large PTVs, but, with smaller PTVs, larger discrepancies were found. Gamma agreement index (GAI) values between 95.5% and 99.6% for all the plans with the threshold criteria 3%/3 mm were achieved, but 2%/2 mm threshold criteria showed larger discrepancies. The TPS algorithm comparison results showed large dose discrepancies in the PTV mean dose $\left(D_{50\%}\right)$, nearly 60%, for the PBC algorithm, and differences of nearly 20% for the AAA, occurring also in the small PTV size range. This work suggests the application of independent plan verification, when the AAA or the AXB algorithm are utilized in lung SBRT having PTVs smaller than 20‐25 cc. The calculated data from this study can be used in converting the SBRT protocols based on type ‘a’ and/or type ‘b’ algorithms for the most recent generation type ‘c’ algorithms, such as the AXB algorithm.

PACS numbers: 87.55.‐x, 87.55.D‐, 87.55.K‐, 87.55.kd, 87.55.Qr

## I. INTRODUCTION

Stereotactic body radiotherapy (SBRT) has attained a status of the treatment technique of choice for malignancies in several anatomical locations. After its first implementation to extracranial treatments reported by Lax et al.^(1)^ in 1994, SBRT has evolved to its present form. The principle of SBRT is based on delivering large doses to planning target volume (PTV) in a single fraction or in a few fractions. Compared to conventional radiotherapy fractionation schemes, this results in higher potential biological effect. In the normal tissue toxicity minimization, the PTV volume plays a key role (i.e., the larger the PTV volume, the larger probability there is for adverse normal tissue complications). Therefore, SBRT is applied only for tumors with diameters not larger than 5 cm, and the size of the PTV has to be minimized. This is achieved with small, patient‐specific target margins, which result from accurate patient positioning and immobilization, and modern image‐guidance. Five to seven or more conformal static treatment fields or arc techniques, such as volumetric‐modulated arc therapy (VMAT), enable rapid dose falloff away from target, which spares the normal tissue around the PTV. The history, development, and application of SBRT has been thoroughly presented in several review articles,^2^, ^3^ clinical trial and protocol reports,^4^, ^5^, ^6^, ^7^, ^8^ and other comprehensive papers^(9)^ by numerous research groups.

One of the major challenges in lung SBRT is the accuracy of the dose calculation. Challenges originate from the issues related to small‐field dosimetry, since measured data are required in dose calculation algorithm configuration. This has consequences, especially with measurement‐based algorithms, whereas with model‐based algorithms the inherent performance of the algorithm defines the achievable accuracy level. In dose calculation, largest inaccuracies occur in areas of steep dose gradients being largest in small fields delivered through heterogeneities having low density, such as the lung tissue. This is due to the inability of dose calculation algorithms to model lateral electron transport, especially in a state of “electronic disequilibrium”. The discrepancies are specific especially to type ‘a’ algorithms, which are usually based on pencil beam convolution principles. More advanced type ‘b’ algorithms, based on superposition convolution principles, produce more accurate results, but the differences are still too large in most complex cases when compared to reference methods.^(3)^ Recently, a next‐generation dose calculation algorithm has been released, for which preliminary results in homogeneous^10^, ^11^ and heterogeneous^12^, ^13^, ^14^, ^15^, ^16^ phantoms are superior when compared to conventional algorithms.

In radiotherapy, full Monte Carlo (MC) simulations are considered to provide dose distributions that can be used as a primary reference for various purposes.^(17)^ There are several studies available exploiting MC methods in benchmarking commercial dose calculation algorithms and, in the case of clinical lung SBRT, plan dose comparisons with full^18^, ^19^ and fast^20^, ^21^, ^22^, ^23^ MC codes have been carried out. These studies have reported dosimetric discrepancies for the minimum dose in PTV to be as large as about 40% for type ‘a’ algorithms and about 10% for type ‘b’ algorithms. However, studies concerning dose calculation accuracy in lung SBRT with the most recent generation of algorithms and full MC simulation have not yet been reported. In the study by Rana et al.^(16)^ a type ‘b’ algorithm was compared to the calculation results of a new generation algorithm in lung SBRT treatments, finding differences up to 10%.

The goal in this work is to compare lung SBRT plan dose distributions obtained with dose calculation algorithms of different generations implemented in Eclipse treatment planning system (TPS) by Varian Medical Systems, Inc. (VMS) (Palo Alto, CA, USA). In TPS algorithm comparison, we used one of the algorithms (the most recent dose calculation algorithm by VMS, Acuros XB (AXB) algorithm) as the baseline for other TPS algorithms. To evaluate the accuracy of the AXB algorithm, we recalculated selected AXB‐calculated plans with the full MC model and compared the dose distributions with gamma analysis, quantitative dose‐volume histogram (DVH) parameters, and dose difference profiles. After assessing the accuracy of the AXB algorithm, we created ten SBRT treatment plans for the lung with pencil beam convolution (PBC) algorithm (type ‘a’) and anisotropic analytical algorithm (AAA) (type ‘b’). We benchmarked the algorithms with respect to DVH parameters of the PTV, ipsilateral lung, and spinal cord by preparing optimal plans using both algorithms and then recalculated the plans with the AXB algorithm (which here is categorized as type ‘c’ algorithm), to allow the mutual comparison. In our study, the comparison has been taken to the next level from the study by Rana et al.^(16)^ by comparing the calculation results to full MC simulation and providing results compared to type ‘a’ algorithm. Another merit of this work is in that, whereas in other earlier studies comparisons are based on calculations and measurements in virtual phantoms, we have performed the full MC‐based comparisons in clinical patient plans, with which measurement‐based verification methods inside patients are impossible to apply.

This study has two motivations. On one hand, the inclusion of the widely decommissioned type ‘a’ algorithm enables the reader to retrospectively link its dosimetric aspects to the analysis of treatment outcomes. Also, since the published data are mostly based on dose calculations with type ‘a’ algorithm, the results of our study contributes to transferring of this data through type ‘b’ algorithm results to most recent type ‘c’ algorithm. On the other hand, this study demonstrates the performance of the new type ‘c’ algorithm for lung SBRT dose calculation with clinical plans. Requests for this kind of study can be found in the literature.^8^, ^11^ At the authors' clinic, the value of the work is self‐evident because, after the commissioning of lung SBRT for clinical use in 1999, for 75% of over 150 patients treated to date, the treatment plans have been calculated with the PBC algorithm (type ‘a’) and the rest with the AAA (type ‘b’). Our investigation on the treatment outcomes, with dosimetric analysis, will take place in the near future, as will also the commissioning of the AXB algorithm (type ‘c’), after comprehensive benchmarking.

## II. MATERIALS AND METHODS

### A. The TPS dose calculation algorithms

In this study, version 10.0 of the Eclipse TPS and version 10.0.28 of the algorithms were used. Commissioning of the algorithms was performed strictly following manufacturer's manuals and recommendations, using the same measurement data for all algorithms, where applicable, and default configuration settings. The smallest field size in output factor configuration was $2\times 2\;{\text{cm}}^{2}$ for all the algorithms and it was smaller than the smallest field size (defined by the jaws) needed in dose calculations. The measurements were performed with IBA SFD DEB050 stereotactic field detector (IBA Dosimetry AB, Sweden) for 2×2 to $4\times 4\;{\text{cm}}^{2}$ fields, and with PTW TM31002 Semiflex ionization chamber (IC) (PTW Freiburg GmbH, Germany) for 3×3 to $40\times 40\;{\text{cm}}^{2}$ fields using a motorized scanning system in an MP3 water phantom (PTW Freiburg GmbH). The output factor measurement results were daisy‐chained, as presented by Dieterich et al.^(24)^ The PBC algorithm is an analytical correction‐based algorithm where the dose is calculated by convoluting the field intensity fluence with narrow pencil beam kernels. Subsequently, corrections for patient surface obliquity and heterogeneities are performed.^25^, ^26^, ^27^ The AAA is a semi‐analytical, model‐based algorithm, although its core is built on exploiting pencil beams. The pencil beams are determined from Monte Carlo simulations, fitted to user‐supplied beam measurements, after which three separate subsources — primary photons, extrafocal photons, and electron contamination — are modeled. Heterogeneity correction in the AAA is partly similar to the one in PBC algorithm, but to some extent, it also takes into account the scattered radiation from the surroundings of the calculation point (i.e., in the lateral scaling of the medium it applies six independent exponential functions to account for the lateral transport of energy with varying densities).^28^, ^29^, ^30^ The AXB algorithm is a nonanalytical model‐based algorithm and represents the most recent generation of clinical dose calculation algorithms. It solves deterministically the coupled system of linear Boltzmann transport equations (LBTEs). It uses the same subsource models as implemented in the AAA, but in the patient dose calculation, the following steps are performed: 1) transport of source model fluence into the patient, 2) calculation of scattered photon fluence in the patient, 3) calculation of scattered electron fluence in the patient, and 4) dose calculation.^(10)^ In heterogeneity correction, the AXB algorithm explicitly models the physical interactions of radiation with matter and, thus, the report mode for the final dose distribution is referred to as dose‐to‐medium in medium $\left(D_{m,m}\right)$. Although the AXB algorithm inherently calculates $D_{m,m}$, the dose distributions can be converted to dose‐to‐water in medium $\left(D_{w,m}\right)$, which is done by replacing the medium‐based fluence‐to‐dose response function used in absorbed dose calculation with a water‐based response function. In the PBC algorithm and in the AAA, the dose report mode is also $D_{w,m}$, but in those algorithms the dose results are based on electron density‐based corrections applied to dose kernels calculated in water.^10^, ^11^, ^13^ Therefore $D_{w,m}$ mode of the AXB algorithm represents more closely true absorbed dose to water.^(12)^ For square fields in field sizes relevant to this study, the TPS algorithms produced dose distributions, compared to TPS beam data measurements, in water with dose differences less than 1.0% in percentage depth dose (PDD) curves at depths beyond depth of dose maximum $\left(d_{\max}\right)$, less than 0.7% in profiles in high‐dose and out‐of‐field regions, and distance‐to‐agreement (DTA) values less than 1.8 mm in the penumbral region (results not presented in this paper).

### B. The MC model

“Full” MC simulations were performed with the BEAMnrc code package (V4‐2.3.1, or BEAMnrc 2010) based on the EGSnrc MC code that simulates coupled electron‐photon transport. The EGSnrc‐based phantom dose calculation is performed with DOSXYZnrc, also included in the BEAMnrc code package.^(31)^ The geometry model of the linear accelerator (linac) treatment head was based on the Varian Clinac iX (2300C/D) linac equipped with Millennium 120 MLC (5 mm thick leaves at isocenter plane around beam central axis (CAX)). The MC model was based on the earlier work by the authors,^(32)^ changing the treatment head components specific to the electron beam mode to ones specific to the photon beam mode used in this study.

The nominal photon beam energy was 6 MV. The simulation of beam generation and beam transport in the treatment head was divided into two phases to allow the absolute dose calibration of the MC model, following the technique by Popescu et al.^(33)^ The initial electron beam of the MC model was selected to be of circular shape with a Gaussian intensity distribution of 0.6 mm FWHM. The initial electron beam energy spectrum at the X‐ray target level was negatively skewed in shape, having a lower energy “tail”, peaking to the energy of 5.9 MeV Other perspectives of the iterative initial electron beam tuning process and beam parameter selection are discussed in Ojala et al.^(34)^ For square fields in field sizes relevant to this study, the MC model produced dose distributions, compared to TPS beam data measurements, in water with dose differences less than 1.0% in PDD curves at depths beyond $d_{\text{max}}$, less than 0.5% in profiles in high‐dose and out‐of‐field regions and DTA values less than 0.2 mm in the penumbral region (results not presented in this paper). In the first phase simulation directional bremsstrahlung splitting (DBS) was applied with a splitting factor of 1000. This simulation had to be performed only once, with the number of particle histories of $10\times 10^{9}$. The resulting particle data were collected into a phase space file, which was used as source in the second phase simulation through beam‐modifying components, for which the number of particle histories was the number of resulted particles in the phase space file recycled five times. The contribution from each treatment field was simulated separately, and the parameters representing field apertures defined by jaws and MLC were exported from the TPS and converted to the form required by the MC code package. The following electron and photon transport cutoff parameters were used in all simulations, also in subsequent DOSXYZnrc simulations: ECUT=AE=0.521 MeV and PCUT=AP=0.01 MeV. The value for ECUT for MC represents the cutoff of total (rest+kinetic) energy of the electron and it was chosen to provide accurate secondary electron transport. The cutoff value for electron kinetic energy for the AXB algorithm was 0.500 MeV (1.011 MeV total energy), which is unmodifiable by the user. Other EGSnrc parameters were the same as in the earlier work by the authors.^(32)^

The phase space data from second phase simulation were used as input for dose calculations in the patient geometry, applying the DOSXYZnrc code. The CT‐based patient phantom geometry was reconstructed from a set of three mm‐thick CT slices, exported from TPS, with the CTCREATE code in DOSXYZnrc. The CT number‐to‐material and density conversion curve were defined using the RMI Gammex 467 Tissue Characterization Phantom (Middleton, WI, USA). The same conversion curve was used in Eclipse TPS. Four different materials (AIR521ICRU, LUNG521ICRU, ICRUTISSUE521ICRU, and ICRPBONE521ICRU) were assigned for patient phantom voxels using the conversion curve, and the PEGS4 cross‐sectional data for the materials were applied in MC dose calculation. In the AXB algorithm, adipose and cartilage tissue were also included in the material assignment table, but since they were not available in PEGS4 cross‐sectional library, the CT number range of ICRUTISSUE521ICRU was extended to the upper limit of LUNG521ICRU and lower limit of ICRPBONE521ICRU in the MC model. The number of particle histories used in each DOSXYZnrc simulation was the number of resulting particles from second phase simulation recycled five times. The calculation grid size for CT‐based patient phantoms was 0.125 cm, which was equivalent to the calculation grid size applied with clinical dose calculation algorithms. With MC model, the dose report mode is $D_{m,m}$. The random nature of particle transport is expressed by determining statistical uncertainties for the calculated dose values for simulated particles and dose values within each voxel. The average latent variances in BEAMnrc simulations were typically about ±0.1%, which were taken into account in the average statistical uncertainty of about 1.0% in voxels with doses values greater than 50% of the maximum dose in patient dose calculations performed with DOSXYZnrc. In general, the simulation parameter selection was performed without compromising the calculation accuracy, which led to long calculation times (several thousands of CPU hours per plan), which was the reason why the MC calculations were applied only to limited number of patients.

### C. Patient selection, dose prescription, treatment planning, and comparison

The criteria for the patient selection were to include patients with varying PTV sizes and tumors not in contact with surrounding high‐density structures (i.e., thoracic or mediastinal wall, diaphragm or large blood vessels). The distance of the gross tumor volume (GTV) to such structures was usually at least 1‐2 cm, being at minimum 3 mm for small parts of some GTVs. Structures were delineated utilizing retrospective respiration‐correlated 4D CT (Philips Brilliance Big Bore CT, Philips Healthcare System, Cleveland, OH, USA) technique, from which maximum intensity projection datasets with 3 mm slice thickness were reconstructed. The dose calculation was performed in the average intensity projection of 4D CT datasets. The dose prescription was according to the Nordic SBRT study group, adapted from the original work published by Lax et al.^(1)^ In this dose prescription protocol, doses to central parts of the PTV are about 50% higher than that prescribed at the periphery of the PTV. The prescription isodose level should be 67% of the dose at normalization point, which normally is close to the isocenter and/or the center of mass of the PTV. The dose planning criteria are described more in detail in Lax et al.^(1)^ The prescribed dose was from 45 to 54 Gy in 3 to 5 fractions, depending on the size of the PTV. The plans used 3D conformal radiotherapy (CRT) technique and included five to nine coplanar nonopposing treatment fields with 6 MV photon beams. MLCs were used in static mode and no wedges were applied.

For all the plans, we extracted clinically relevant, quantitative dose‐volume histogram (DVH) parameters for the PTV ($D_{95\%},D_{50\%}$, and $D_{5\%}$; percentage of the dose for which there is 95%/50%/5% volume coverage), for the ipsilateral lung with the GTV subtracted ($V_{30\%}$; percentage of the structure volume for which the percentage dose is 30% or less), and for the spinal cord ($D_{\max}$; percentage maximum dose).^(7)^ To quantify the low dose spillage in the lung around the PTV, the maximum dose at a distance of 2 cm from the PTV $\left(D_{2\text{cm}}[\%]\right)$ was determined.^(7)^ All the parameters were adapted from RTOG 0915 protocol^(7)^ and they were normalized to the prescribed dose. From DVH parameters, also a conformity index (CI)^(35)^ was calculated. It describes how the PTV is encompassed by the prescription dose, but also takes into account how much high‐dose levels are spilling to the surrounding normal tissue around PTV. CI is calculated as follows:

(1) $$CI=\frac{V_{PTV67\%}}{V_{PTV}}\frac{V_{PTV67\%}}{V_{T}}$$

where $V_{PTV67\%}$ represents the volume of PTV receiving the prescription dose, $V_{PTV}$ is the volume of PTV, and $V_{T}$ is total volume receiving the prescription dose.

#### C.1 The AXB algorithm vs. the full MC model

In the first stage of the study, we selected four patients to cover the whole PTV size range from all the treated lung SBRT patients at the authors' clinic: a patient with a small PTV of 4.2 cc, two patients with medium‐sized PTVs of 15.1 cc and 25.6 cc, and a patient with a large PTV of 100.4 cc. Using the selected patient CT datasets, the AXB algorithm was used for treatment planning (dose report mode: $D_{m,m}$), creating the plans to meet the planning criteria. The accuracy of the AXB algorithm was assessed by recalculating the plans with the MC model with the same number of monitor units (MUs) and identical field settings. The AXB‐calculated plans, including the CT datasets, structure sets, and dose distributions, were exported to CERR software, where also the MC‐calculated dose distributions were imported. CERR, which uses MATLAB software (The MathWorks, Natick, MA; version R2011b in this study), is a software package developed at Washington University for the review and analysis of mainly radiotherapy planning data.^36^, ^37^ In addition to DVH analysis including aforementioned DVH parameters, a 3D gamma analysis tool was applied to quantify the accuracy of the AXB algorithm. There were two levels of threshold criteria set for both parameters in the gamma analysis calculation: 2% (of maximum dose) in dose difference, 2 mm in distance‐to‐agreement (DTA) (2%/2 mm) and 3%/3 mm. To minimize the effect of inherent noise present in MC‐based dose distributions on gamma analysis results, large numbers of particle histories were simulated in MC calculations to minimize the statistical uncertainty, and regions with less than 15% of maximum dose were neglected in the gamma analysis calculation. The results were presented with the gamma agreement index (GAI), which is the ratio of the number of calculation points passing the gamma test and the number of all calculation points.

#### C.2 The performance of the TPS algorithms

In the second stage of the study, a subgroup of ten patients (including four patients used in the AXB algorithm benchmark) was selected for this study. Separate plans applying both the PBC algorithm and the AAA for all patients were created to meet the planning criteria, keeping the number of fields and field directions similar but changing only the jaw and MLC apertures. Finally, the plans using the PBC algorithm and the AAA were recalculated with the same number of MUs and identical field settings with the AXB algorithm (dose report mode: $D_{w,m}$). The dose distributions were compared using the aforementioned DVH parameters, $D_{2\text{cm}}$ and CI.

## III. RESULTS

### A. The AXB algorithm vs. the full MC model

The results for the 3D gamma analysis and for the DVH parameter comparison for the accuracy assessment of the AXB algorithm against the MC model are presented in Table 1. For the four plans, the GAI with the threshold criteria of 3%/3 mm was between 95.5% and 99.6%. The threshold criteria of 2%/2 mm revealed more differences. The largest discrepancy, 83.6%, was produced by the plan with the PTV of 15.1 cc. In DVH parameters, the largest discrepancies occurred in the plan with the same PTV; the difference in DVH parameters related to the PTV was at largest 11.9% in $D_{50\%}$. For all the plans, the discrepancies in the $V_{30\%}$ and spinal cord $D_{\text{max}}$ parameters were small. The largest difference in the CIs was 0.13 for the plan with the PTV of 15.1 cc, while with the rest of the plans, the differences were negligible. The results for two plans with largest PTVs showed better overall congruence, as may also be seen in the GAI values. Differences less than 1% can be ignored, since the statistical uncertainties related to the MC‐calculated dose distributions were of the same order.

**Figure 1:**
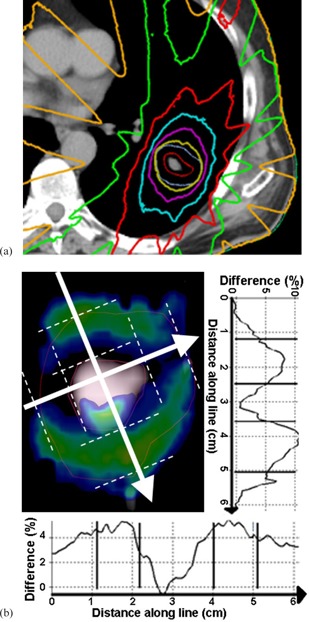
The isodose distribution (a) calculated by the MC model for the plan with medium‐sized PTV (15.1 cc) in transversal isocenter plane. Dose levels from the outermost isodose curve to the innermost one are 5 Gy, 15 Gy, 25 Gy, 35 Gy, 45 Gy (the prescription dose), and 55 Gy. The two innermost contours represent the PTV and the GTV, respectively. A sagittal isocenter plane (b) showing colored areas, where the gamma calculation typically failed (threshold criteria 3%/3 mm). The graphs along the lines show the percentage dose difference between the MC‐recalculated AXB‐plan and the original AXB‐plan through the largest dose differences in orthogonal directions. Lines in the profiles represent the PTV and the GTV boundaries.

Figure 1 presents isodose distribution (Fig. 1(a)) and positional distribution of the dose discrepancies (Fig. 1(b)) for the plan with the medium‐sized PTV of 15.1 cc. In the dose difference graphs (Fig. 1(b)), dose values in the cranio–caudal direction are nearly 11% larger with the MC model than with the AXB algorithm, located in the low‐density areas in the lung around high‐density tumor in the cranial and caudal corners of the PTV margin. In the transversal direction, the largest differences occur also in the PTV margin, but are half, about 5% of the difference in the other direction. In the central PTV, the dose values for the MC model are about 0% to 3% larger than for the AXB algorithm.

**Table 1:** Comparison of the results between the dose distributions from original AXB‐calculated plans which were recalculated with the MC model. All DVH parameter results represent the difference values between the original AXB‐calculated plan and the MC‐recalculated AXB‐plan. Absolute dose values for various DVH parameters from different plans are normalized to the prescribed dose values

	*Patient #1*	*Patient #2*	*Patient #3*	*Patient #4*
PTV volume (cc)	4.2	15.1	25.6	100.4
*GA*	*(%)*	*(%)*	*(%)*	*(%)*
3%/3 mm	98.9	95.5	99.4	99.6
2%/2 mm	92.6	83.6	94.4	95.2
*Parameters*		*Difference (%)*		
Lung				
$D_{2\text{cm}}$	3.3	−7.6	−2.7	−1.4
PTV				
$D_{95\%}$	−4.0	−11.1	−3.6	−1.8
$D_{50\%}$	−7.3	−11.9	−4.4	−1.8
$D_{5\%}$	−10.2	−9.4	−1.6	−0.2
Lung‐GTV				
$V_{30\%}$	−0.6	−1.5	−0.5	−0.2
Spinal cord				
$D_{\max}$	−0.2	0.0	1.3	1.0
CI (absolute diff.)	0.03	0.13	0.05	0.01

### B. The performance of the TPS algorithms

**Figure 2:**
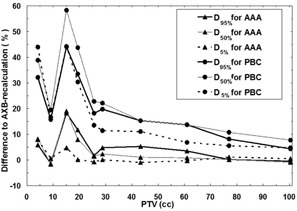
Comparison of the results for the algorithms as a function of the PTV size. Lines with triangles represent the differences for $D_{95\%},D_{50\%}$, and $D_{5\%}$ between the AAA and the AXB‐recalculated AAA plan, and lines with circles represent the differences for $D_{95\%},D_{50\%}$, and $D_{5\%}$ between the PBC algorithm and the AXB‐recalculated PBC plan. Solid lines are for $D_{95\%}$, grey lines for $D_{50\%}$, and dashed lines for $D_{5\%}$.

The results of the TPS algorithm comparison are presented in Table 2 and in 2, 5. Certain DVH parameters show large dosimetric uncertainties for the smallest PTV volumes. For DVH parameters related to the PTV $\left(D_{95\%},D_{50\%},D_{5\%}\right)$ with both the PBC algorithm and the AAA the differences compared to AXB‐recalculated dose distributions become larger with the decreasing size of the PTV, which is also visualized in Fig. 2. Also, the PBC algorithm produces larger discrepancies than the AAA throughout the PTV size range. The largest discrepancies between the PBC and AXB algorithms, and the AAA and the AXB algorithms in $D_{50\%}$ are 58.3% and 19.1%, respectively, which both occur in a plan with the PTV size of 15.1 cc. The largest discrepancies are located in the cranial and caudal parts of the PTV margin, where the PBC algorithm and the AAA consistently produce larger dose values than present in the AXB‐recalculated plans.

**Table 2:** Comparison of the results between dose distributions from original PBC‐ and AAA‐based plans, which were recalculated with the AXB algorithm. All DVH parameter results represent the difference values between the original AAA‐calculated plan and the AXB‐recalculated AAA plan or the original PBC‐calculated plan and AXB‐recalculated PBC plan. Absolute dose values for various DVH parameters from different plans are normalized to the prescribed dose values

	*Maximum Difference (%)*		*Minimum Difference (%)*		*Mean Difference (%)*	
*Parameters*	*AAA*	*PBC*	*AAA*	*PBC*	*AAA*	*PBC*
Lung						
$D_{2\text{cm}}$	6.5	25.7	0.4	3.3	0.8	11.2
PTV						
$D_{95\%}$	18.3	44.1	0.2	4.4	5.0	20.5
$D_{50\%}$	19.1	58.3	0.2	7.8	3.9	25.3
$D_{5\%}$	8.0	44.3	0.2	4.8	1.4	18.9
Lung‐GTV						
$V_{30\%}$	1.6	3.8	0.0	0.0	0.3	1.2
Spinal cord						
$D_{\max}$	−1.4	3.1	0.0	0.0	0.1	0.4
CI (absolute diff.)	0.25	0.58	0.00	0.00	0.03	0.16

**Figure 3:**
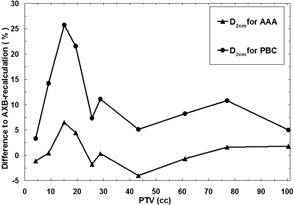
Comparison of the results for the algorithms as a function of the PTV size. Solid line with triangles represents the difference in $D_{2\text{cm}}$ between the AAA and the AXB‐recalculated AAA plan, and solid line with circles represents the differences in $D_{2\text{cm}}$ between the PBC algorithm and the AXB‐recalculated PBC plan.

The parameter $D_{2\text{cm}}$ showed also larger dose deviations for the small‐sized PTVs, which is visualized in Fig. 3. The dose differences at a distance from the PTV are more pronounced with the smaller sized PTVs, while with the two smallest PTVs the discrepancies decrease. The largest discrepancies between the PBC and AXB algorithms and the AAA and the AXB algorithm were 25.7% and 6.5%, respectively, and they occurred in a plan with the PTV of 15.1 cc.

**Figure 4:**
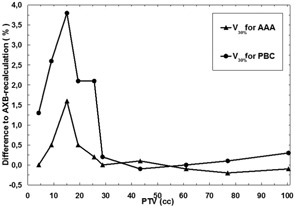
Comparison of the results for the algorithms as a function of the PTV size. Solid line with triangles represents the difference in $V_{30\%}$ for the ipsilateral lung with the GTV subtracted between the AAA and the AXB‐recalculated AAA plan, and solid line with circles represents the difference for the same DVH parameter between the PBC algorithm and the AXB‐recalculated PBC plan.

In the lung, the dose differences are smaller in general, when compared to discrepancies in the PTV‐related DVH parameters and in $D_{2\text{cm}}$. Only in plans with smallest PTVs, the $V_{30\%}$ DVH parameter shows considerable dose differences, being maximally 3.8% with the PBC algorithm and about 1.6% with the AAA. The mean values for the differences with both algorithms were small, although slightly larger for the PBC algorithm. The differences for the $V_{30\%}$ are visualized in Fig. 4.

**Figure 5:**
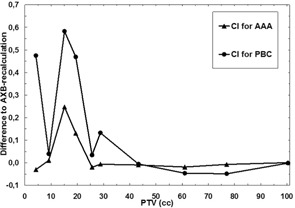
Comparison of the results for the algorithms as a function of the PTV size. Solid line with triangles represents the difference in CI between the AAA and the AXB‐recalculated AAA plan, and solid line with circles represents the differences for the CI between the PBC algorithm and the AXB‐recalculated PBC plan. The reader should note the absolute scale used.

For the spinal cord, no dependence on the size of PTV was found and the maximum and mean values for the differences were comparable to the results for the $V_{30\%}$ parameter, with the maximum difference for the PBC algorithm being 3.1% and 1.4% for the AAA, while the mean differences were negligible.

The achieved values for CI for different plans in this study ranged from 0.60 to 0.80, values closer to 0.60 for the plans with small PTVs and values closer to 0.80 for the plans with large PTVs (not shown). The differences in CI (Fig. 5) show similar behavior as a function of the size of the PTV, as do DVH parameters for the PTV, $V_{30\%}$ and $D_{2\text{cm}}$. With the PBC algorithm, the largest absolute differences are more than twice larger than with the AAA and with both the differences increase with decreasing size of the PTV in the plan. The relative discrepancies are drastic, especially for the PBC algorithm, for which the value for CI was nearly thirteenfold (0.63 vs. 0.05) that obtained for the AXB‐recalculated PBC‐plan in the plan with PTV size of 15.1 cc. For this plan, the CI for the AAA was only 1.5‐fold (0.70 vs. 0.46), when compared to the AXB‐recalculated AAA‐plan.

The computation times for all the plans were in the range from 24 sec to 30 sec, 29 sec to 43 sec and 3 min 57 sec to 4 min 13 sec for the PBC algorithm, the AAA, and the AXB algorithm, respectively. In the calculations, the distributed calculation framework (DCF) of the Eclipse TPS was used. In each calculation, the DCF distributed one field per calculation node. In the AXB algorithm calculations, “the field dose” option was selected.

## IV. DISCUSSION

### A. The AXB algorithm vs. the full MC model

The results for the comparison between the AXB algorithm and the full MC model indicate that the AXB algorithm can be used as a baseline for the other two TPS algorithms with reserve. In gamma analysis applied to plan verification, which is usually performed in 2D, the threshold criteria is very often set to 3%/3 mm, which is also recommended by the American Association of Physicists in Medicine (AAPM) Task Group Report TG‐119^(38)^ for the quality assurance of intensity‐modulated radiotherapy (IMRT) that contains fields with large dose gradients produced by small field segments. In this study, the gamma analysis with aforementioned threshold criteria was performed in 3D, for which the achieved agreement levels of higher than 95.5% for all four plans suggests that the differences between the AXB algorithm and the MC model are clinically acceptable. The more stringent threshold criteria of 2%/2 mm reveals differences increasing in an expected manner (i.e., with the decreasing size of the PTV) and especially for the plan with the PTV of 15.1 cc, for which the largest discrepancies occurred also between the TPS algorithms.

In the DVH parameters, the differences between the AXB algorithm and the MC model showed the largest discrepancies in the dose calculation for the mean $\left(D_{50\%}\right)$ and near minimum $\left(D_{95\%}\right)$ dose values of the PTV, except for the plan with the smallest PTV, for which the largest discrepancy occurred in areas of near maximum doses $\left(D_{5\%}\right)$. The largest differences occurring in the $D_{50\%}$ and $D_{95\%}$ parameters imply that the discrepancies occur in the PTV margin, which is demonstrated in Fig. 1(b). With decreasing PTV size, the proportion of the margin volume increases relative to the total PTV volume and, therefore, these discrepancies become larger in plans with decreasing PTV size. In addition to inherent sources of systematic error in the AXB algorithm due to discretization of solution variables, as described in Failla et al.,^(10)^ a presumable explanation for aforementioned differences is the considerably higher electron transport energy cutoff value used in the AXB algorithm (1.011 MeV) compared to the value used with the MC model (0.521 MeV). This leads to the lower dose values in the PTV margin towards the periphery of the PTV in the AXB‐calculated dose distributions. The difference is largest near the field edge and the contributions from the fields accumulate to the same part of the PTV margin in the cranial and caudal ends of the PTV. In the other parts of the PTV margin the difference is partially compensated by central parts of the other fields.

Contribution for the difference may also arise from how different materials are assigned in the AXB algorithm and in the MC model calculations in this study. The density of the material in the PTV margin is less than unity, falling steeply to density ranges of adipose and lung tissues. In the AXB algorithm, the upper limit of the density range of the adipose tissue extends nearly to unity, contrary to the MC model calculations. With the MC model, the adipose tissue is replaced by extending the density range of the skeletal muscle (ICRUTISSUE521ICRU) down to the upper limit of the lung tissue, which is the same as in the AXB algorithm. In the plan with the smallest PTV, the HU values of the GTV in the average intensity projection CT dataset fall into range of the adipose and the lung tissue limit, which is due to the blurred HU values of the very small tumor moving with respiration. This is contrary to the much higher density GTVs in the centers of PTVs in the other plans. The increased contribution of differences in material assignments between the calculations, alongside the different cutoff energies, might be the reason for the largest discrepancy occurring in the areas of highest doses $\left(D_{5\%}\right)$ in the plan with the smallest PTV.

### B. The performance of the TPS algorithms

In plan comparison assessing the accuracy of the AXB algorithm using the MC model, the dose report mode was $D_{m,m}$, which is an inherent default option for both calculation methods. This was the motive to perform the comparison using $D_{m,m}$ mode, whereas, in TPS algorithm comparison, the dose report mode $D_{w,m}$ of the AXB algorithm was used to allow the mutual comparison of the AXB algorithm to the AAA and the PBC algorithm. Since the purpose of this study was to assess the performance and reveal discrepancies between different algorithms in clinical practice, setting the dose report mode to the same was a natural choice for a fair comparison. The accuracy of the AXB algorithm in heterogeneous media using both dose report modes in other contexts than SBRT has been assessed.^12^, ^13^, ^14^, ^15^

The comparison between the TPS algorithms showed levels of discrepancies that are likely to influence the treatment outcomes. According to Report of AAPM Task Group No. 105, dose differences as low as 5%‐10% are reported to be clinically detectable, and may result in 10%‐20% changes in tumor control probability or 20%‐30% changes in normal tissue complication probability.^(17)^ For the PTV, the DVH parameters differed nearly 60% between the PBC algorithm and the AXB algorithm for the plan with the most central PTV of 15.1 cc with a distance of at least 2 cm from the surrounding high‐density structures. The overall performance of the PBC algorithm decreased considerably when the sizes of the PTVs fell below 25 cc. The differences from the AXB‐recalculated dose distributions were larger than 5% for the whole PTV size range. The AAA showed discrepancies less than 5% for the PTV size range down to 25 cc, being maximally less than 20% for the plan with the most central PTV of 15.1 cc. From the viewpoint of tumor control, the results indicate the known fact that the PBC algorithm should not be used in the clinical dose calculation for the lung SBRT. The discrepancies between the AAA and the AXB algorithm with plans having PTVs smaller than 20‐25 cc, in connection with discrepancies between the MC model and the AXB algorithm, imply that the plans need further verification prior to treatment (e.g., against full MC dose calculation and/or measurements).

For the $D_{2\text{cm}}$ parameter characterizing the dose spillage to the surroundings of the PTV, both the PBC algorithm and the AAA perform better than with the DVH parameters related to the PTV, although the maximum discrepancies still are at an unacceptable level. The mean deviation of $D_{2\text{cm}}$ was 11.2% for the PBC algorithm and only 0.8% for the AAA, indicating that the heterogeneity correction applied in the AAA is able to predict the dose much more accurately than the PBC algorithm. For the $V_{30\%}$ parameter, the maximum differences were 3.8% and 1.6%, and mean differences were 1.2% and 0.3%, for the PBC algorithm and the AAA, respectively. The reason for these relatively small discrepancies is that the volume of the healthy lung tissue through which the beams travel is small compared to the volume of the whole lung. The discrepancies for the spinal cord were even smaller, which is because the absolute dose levels in spinal cord were very low, since it is avoided in the treatment planning. The last parameter describing the discrepancies between different algorithms was CI, which combines the PTV coverage and the dose spillage to the surroundings of the PTV. The CI is a step to a more clinically‐relevant quality parameter for plan comparison purposes, especially applicable to SBRT, where extremely high conformality with high‐dose gradients and minimal coverage in the healthy tissue is sought. The results revealed how large differences may occur in lung SBRT with small‐size PTVs between dose calculation algorithms from three generations and attests also to the applicability of the CI to represent a sensitive quality parameter for SBRT plan comparison.

The intention of this study was to choose plans with tumors not in contact with the surrounding high‐density anatomical structures to minimize the contribution of scattered radiation from these structures to dose distributions and, thus, to enable a fair comparison between the plans. Regardless of that, we noticed that the differences in the DVH parameters are sensitive to the anatomical location of the PTV. Varying distances of higher density tissues affected the quantitative DVH parameter values of the PTVs insomuch that the dependence of the calculation differences on the size of PTV was compromised. An example of this behavior can be seen with the PTV of 9.1 cc (2, 5), where the PTV locates centrally in the lung, but the distance to the posterior thoracic wall and to the caudal end of the lung are small. As a consequence, the discrepancies for both the PBC algorithm and the AAA are notably smaller than those of the other plans in the same PTV size range. On the contrary, in the plan with the PTV of 15.1 cc, for which the largest deviations occurred, the PTV was located the most centrally of all the studied patient cases and additional difficulty for the dose calculation was introduced by the complex‐shaped bronchus located partially inside the PTV. As a conclusion, it is difficult to find an explicit relation for the location of a PTV. The similar behavior of the parameters can also be seen in a comprehensive comparison for the type ‘a’ and type ‘b’ algorithms by Hurkmans et al.^(8)^ The results in this study represent mostly the worst case scenario of the discrepancies between the algorithms concerning small targets locating centrally in the lung.

The purpose of this study was not to show the inapplicability of the PBC and other type ‘a’ algorithms for lung SBRT dose calculation, which has been shown by numerous research groups and discouraged by the most recent clinical trial and protocol reports.^6^, ^7^, ^8^ The intention was firstly to produce dosimetric data for the radiotherapy community to transfer dose prescription and normal tissue tolerance protocols based on type ‘a’ algorithm calculations for type ‘b’ dose calculation algorithms, such as the AAA, and secondly, to aid the community further in converting the SBRT protocols for the most recent type ‘c’ algorithm. This study also demonstrates that, until general conclusions on the improved accuracy of a new dose calculation algorithm over previous generation algorithms can be drawn, extensive benchmarking also with clinical patient datasets including various anatomical sites and treatment techniques has to be performed, albeit other studies using homogeneous and heterogeneous virtual phantoms have shown excellent results. In the light of this study, further development is needed to achieve even better congruence between the AXB algorithm and the MC model. This requires an access to the most recent version of the AXB algorithm (version 11), where enhancements to the low‐energy electron transport and low‐density material assignment have been incorporated.^(14)^ We aim to extend the scope of our investigation to MC‐based VMAT SBRT.

## V. CONCLUSIONS

The accuracy of a type ‘c’, the AXB algorithm, in dose report mode $D_{m,m}$, was assessed against full MC simulations for clinical lung SBRT plans with varying sized centrally located PTVs. We observed high level of agreement, especially with 3D gamma analysis. The AXB algorithm, in dose report mode $D_{w,m}$, was used as the baseline in TPS algorithm comparison against a type ‘a’, the PBC algorithm, and a type ‘b’ algorithm, the AAA. The results showed large discrepancies for the PBC algorithm and notable differences between the AAA and the AXB algorithm, especially for plans with small PTVs. Some results even implied that the AAA produced dose distributions closer to the MC model than the AXB algorithm. This work encourages the application of further plan verification methods, such as full MC dose calculation and/or measurements, when the AAA or the AXB algorithm are used in lung SBRT having PTVs smaller than 20‐25 cc. Also more comprehensive benchmarking of the version 10.0.28 of the AXB algorithm is needed prior to clinical commissioning. However, the calculated data from this study, in addition to further contribution from scientific community in the form of similar studies, can be used in converting the SBRT protocols based on type ‘a’ and/or type ‘b’ algorithms for the most recent generation type ‘c’ algorithms, such as the AXB algorithm.

## ACKNOWLEDGMENTS

This study was financially supported by the legacy (allotted to the development of cancer treatment) of Seppo Nieminen.
